# Assessment of COVID-19 prevention practice and associated factors in Jimma town, Ethiopia: A mixed study

**DOI:** 10.3389/fpubh.2022.950202

**Published:** 2022-09-26

**Authors:** Deriba Bedane, Daba Abdissa, Bati Leta, Urge Gerema, Abraham Lomboro, Guta Kune, Abiru Neme, Kumsa Kene, Nimona Berhanu, Abebe Dukessa Dubiwak, Kasahun Girma Tareke

**Affiliations:** ^1^Department of Biomedical Sciences, Faculty of Medical Sciences, Institute of Health, Jimma University, Jimma, Ethiopia; ^2^Department of Epidemiology, Faculty of Public Health, Institute of Health, Jimma University, Jimma, Ethiopia; ^3^School of Nursing, Faculty of Health Sciences, Institute of Health, Jimma University, Jimma, Ethiopia; ^4^School of Pharmacy, Faculty of Health Sciences, Institute of Health, Jimma University, Jimma, Ethiopia; ^5^Department of Health, Behavior and Society, Faculty of Public Health, Institute of Health, Jimma University, Jimma, Ethiopia

**Keywords:** COVID-19, prevention practice, pandemic fatigue, Jimma, Ethiopia

## Abstract

**Background:**

COVID-19 has affected the mental and physical wellbeing, social structure, countries' economy as well as individuals and community resilience, trust, and inequalities among societies. However, now almost all of the activities have been returned to the pre-corona era, despite the emergence of new strains and the spread of the disease. Hence, this study was conducted to assess COVID-19 prevention practice and the associated factors.

**Materials and methods:**

A community-based cross-sectional study triangulated with the qualitative findings was conducted in Jimma town, Oromia, Ethiopia. A total of 422 sample households were involved in the quantitative study. The quantitative data were collected using a structured questionnaire and 12 key informants were also interviewed for the qualitative part. The quantitative data were processed and entered into the Epi Data version 4.6 (software) and analyzed using SPSS 26.0. Similarly, the qualitative data were analyzed using ATLASti.7.1.04 software package. Descriptive statistics and binary logistics regression (*p* < 0.25) were conducted to identify the candidate variable for multivariable logistics regression analysis (*p* < 0.05) and a 95% confidence interval was used to establish the level of significance of the variables with the practice.

**Results:**

Interviews were conducted with a total of 422 participants, yielding a response rate of 100%. Good preventive practices were found to be adopted by 13.3% of the respondents. People aged ≥ 50 years, [AOR = 2.85, 95%, CI = 1.246–0.53] who recovered from COVID-19, [AOR = 2.41, 95%, CI = 1.184–0.92], had chronic diseases [AOR = 3.70, 95%, CI = 1.887–0.25], and living with COVID-19 high risk [AOR = 2.96, 95%, CI = 1.475–0.991 were independently associated with good preventive practices.

**Conclusion:**

In this study, it was understood that there were poor COVID-19 preventive practices among the study participants. There was a disparity in adherence to the preventive practices in relation to (i.e., 50 and above years) the experience of contracting COVID-19 and people aged above 65 years old living with the high-risk group. In addition, the community had different misconceptions or risk perceptions related to COVID-19 infection and preventive practices. This highlights the need to design health education programs and implement risk and/or social and behavior change communication interventions to change perceptions or misconceptions of people or community members to bring about the desired behavioral change and prevent the spread of COVID-19.

## Background

The worldwide health catastrophe of our time and the greatest threat to humanity since World War II is the COVID-19 pandemic. This pandemic is not only a health crisis, but it also has a major impact on daily life, leading to a socio-economic crisis. The pandemic involved every country and triggered a devastating social, economic, and political impact, leaving deep and lasting scars (1, 2).

People did not keep social distance, wash their hands frequently, or wear masks in public until the year 2020. These precautions are now being advised to everyone by health professionals to stop the spread of COVID-19, which is very challenging (3). There have been reports of COVID-19 pandemic fatigue in many nations, which is manifested by a rise in the number of people who did not adhere to guidelines and restrictions to the letter, showing a drop in information seeking, and lower risk perceptions (4, 5). This resulted in a spike in COVID-19 transmission rates, which subsequently increased morbidity and death as well as the risk of re-infection for those who have already contracted the virus (5, 6).

The COVID-19 pandemic is spreading rapidly, with devastating effects on the already fragile livelihoods and unstable economy of sub-Saharan Africa. In addition, as countries face a shortage of oxygen and intensive care unit beds, the number of hospitalizations is increasing rapidly, and the number of deaths is also rising (7, 8).

As a result of misconceptions about the COVID-19 pandemic, such as the denial of its existence, political propaganda, perceiving it as a disease of the rich distorted people's perception, and so on, people did not adhere to standard COVID-19 protective measures to a sufficient degree. Due to these misunderstandings, fewer people followed the advised safeguards, which inevitably raised the likelihood of COVID-19 transmission and the ongoing propagation of misleading information (9–11). The fight against COVID-19 continues on a global scale. The COVID-19 pandemic has been unstoppable so far, and the population remains vulnerable. Although the distribution of the COVID-19 vaccine is still in progress, the emergence of a new coronavirus strain might make the pandemic worse before it improves. New strains of the COVID-19 pandemic have been documented across countries, and they are more virulent and contagious (12, 13). In spite of this threat, countries like the Middle East, Russia, Africa, and a few European nations have reported low rates of COVID-19 vaccination adoption and coverage. This may also pose a significant obstacle to the efforts being made around the world to fight the COVID-19 pandemic (14). Therefore, evaluating preventive practices is the most important way to deal with the spread of COVID-19. Furthermore, understanding COVID-19 prevention practices and related factors are the basis for implementing strategies to prevent such incidents. Hence, this study aimed to assess COVID-19 prevention practices and associated factors in Jimma town, Southwest Ethiopia.

## Methods and materials

### Study setting

A community-based cross-sectional study triangulated with the qualitative findings was conducted from 1st to 30th April 2021 in Jimma town, Oromia regional state, Ethiopia. Jimma town is located around 352 km southwest of Addis Ababa. The town had 17 kebeles. Two public hospitals, Jimma Medical Center and the Shanan Gibe Hospital were used to serve patients as COVID-19 treatment centers.

### Study population and eligibility

All adult (≥18 years) population living in Jimma town within the past 6 months before the data collection date was used as a source population. The study population included all the randomly selected heads (representative) of the households. All heads or representatives of the households who were unable to speak and/or hear, individuals with serious mental illness or severely sick, or who lived in the study area for less than 6 months were excluded from the study.

### Sample size determination and sampling procedure

The sample size was determined using a single population proportion formula by considering *p* = 50%, the margin of error = 5%, and a 95% confidence interval. $n\;=\;\frac{{\left(z\frac{\alpha }{2}\right)}^{2}p\left(1-p\right)}{d^{2}}\;=\;\frac{{\left(1.96\right)}^{2}\times 0.5\;\times \;0.5}{{\left(0.05\right)}^{2}}\;=\;384.$ Adding a 10% non-response rate (384 + 38), the final sample size was calculated to be 422.

Out of the 17 kebeles in Jimma town, five kebeles (≈30%) were selected randomly. Then, the total sample was allocated proportionally to selected kebeles (Table 1). Similarly, the sample size was allocated to each randomly selected gott in the kebele. Systematic random sampling was employed in a quantitative study and the sampling interval was determined based on the total number of households in each selected gott. Households were the primary sampling unit, and the heads of the households were the people who were chosen to be interviewed. When the household heads were unavailable, another member of the family who could adequately respond to the questions was interviewed.

**Table 1:** Proportional allocation of a sample size to randomly selected Kebeles in Jimma Town, Ethiopia, 2021.

**Selected kebele**	**Number of HHs**	**Proportionally**
		**allocated samples**
Bacho bore	6,971	153
Ginjo guduru	3,843	84
Mentina merkato	2,778	61
Bossa addis	2,500	55
Awetu mendera	3,148	69
Total sample size		422

For the qualitative study, a total of 12 key informant interviews were conducted with purposefully recruited health professionals, religious leaders, kebele leaders, community members, and teachers and students.

### Data collection tools and procedure

#### Data collection tools

The questionnaire was adopted from World Health Organization (WHO) framework for supporting COVID-19 pandemic prevention practices (5). The respondents' prevention practices were assessed within the previous 7 days preceding the survey. This tool has three parts (namely sociodemographic, COVID-19 personal experiences, and COVID-19 prevention practices). The COVID-19 prevention practices were measured by 15 items of Likert scale questions (never, rarely, sometimes, often, and always) with a minimum score of 15 and a maximum score of 75. These items include, hands should be washed with soap and water frequently, a sanitizer-based cleaner should be used on hand in case there is no water or soap; when coughing or sneezing, a tissue or the inner hand should be used; mouth, nose, or eyes should not be touched with an unclean hand. A distance of at least 1 m should be maintained between one individual and the next; social gatherings should be avoided, a face mask should be worn, and the face mask should be disposed of appropriately, equipment should be cleaned, precaution should be taken while shopping, and restrictions should be obeyed. Finally, for the regression analysis and discussion, the COVID-19 preventive practice was classified as “good preventive practices” or “poor preventive practices.”

For a qualitative study, data were collected using a semi-structured guide which was developed in the English language and then translated into Afan Oromo and Amharic languages and back-translated into English by an independent translator. The guides were prepared with the research questions starting from general and moving to specific taking into consideration the local knowledge and cultural sensitivities. It was developed to address the community's perception and experience toward COVID-19 and its preventive practices. It contained 6–8 guiding questions that were developed and customized according to the type of respondent.

#### Data collection procedure

The data were collected by seven data collectors (four BSc in Nursing and three in Public Health) and supervised by two MPH professionals. The quantitative data were collected after face-to-face interviews with the study participants. The qualitative data were collected in the participant's natural setting. Three persons (Kasahun Girma Tareke, Deriba Bedane, and Daba Abdissa) conducted/facilitated the key informant interviews using guiding questions and raising probing questions to cover all relevant topics. Note and voice of respondents were audio-recorded. At the beginning of the interviews, participants were informed of the study's objective and the topic for the discussions in detail, and then they provided written informed consent on an individual basis to participate in the study and have their voices recorded. On average, the interviews lasted from 25:00 to 45:15 min.

### Study variables

The dependent variable of this study was COVID-19 prevention practice. The independent variables were sociodemographic factors (age, sex, educational status, occupation, marital status, monthly income, family size, and living with a high-risk population age group), factors related to COVID-19 personal experience (own or someone close), and vaccination for COVID-19.

### Operational definition

#### COVID-19 preventive practices

Refers to the respondents' preventive activities that are recommended by the WHO and Ministry of Health (MOH) to reduce the spread of COVID-19 within the previous 7 days preceding the survey. It was measured by 15 Likert scale questions (never, rarely, sometimes, often, and always) with a minimum score of 15 and a maximum score of 75.

#### Poor COVID-19 prevention practices

A respondent who scored less than the 75% of the sum score of the Likert scale questions.

#### Good COVID-19 prevention practices

A respondent who scored 75% and above on the sum score of the Likert scale questions.

#### Chronic diseases

A participant with a known diagnosed disease, such as diabetes, hypertension, asthma, or chronic kidney diseases.

#### Kebele

The smallest administrative unit in Ethiopia.

#### Gott

A subdivision of kebele into three neighborhoods.

### Data management and statistical analysis

After checking for completeness, the data were entered into Epi-data version 4.6 and exported to SPSS version 26 for analysis. Outliers and missing values were investigated in the exported data. Cross-tabulations and descriptive statistics, such as frequencies, percentages, means, and standard deviations were calculated. To select variables for multivariable logistic regression analysis, binary logistic regression analyses were performed. Candidates for multivariable logistic regression analysis were variables with a *p*-value of 0.25 in binary logistic regression. After that, a multivariable logistic regression analysis was performed. The adjusted odds ratio (AOR) with a respective 95% confidence interval (CI) and a *p*-value of less than 0.05 was used to declare statistically significant COVID-19 prevention practice determinants.

The qualitative data were analyzed using the atlas ti.7.1.04. The data were transcribed verbatim and translated to English to carry out the analysis. Reading and re-reading of the data were carried out to understand and extract the important concepts from the data, and line-by-line coding was done. Then, the findings were presented by triangulating with the quantitative findings supported by quote (s).

### Data quality assurance

To ensure consistency, the questionnaire was blindly translated from English to Afan Oromo and Amharic and back to English. Data collectors and supervisors received 3 days of training on the purpose of the study and data collection procedures. To assure accuracy and comprehensiveness, a pre-test on 5% of the total sample size was conducted in Agaro town prior to the real data collection. Based on the results of the pre-test, modifications and corrections were made. The collected data were checked for completeness, accuracy, clarity, and consistency on a daily basis.

The trustworthiness of the qualitative findings was ensured through different techniques. First, a semi-structured questionnaire was developed concerning the research questions. Second, a diversified group of individuals was recruited from different study settings. Third, peer debriefing was done daily. Fourth, member checking was done with key study participants to check the interpretations of the findings, and their critiques or comments were incorporated. Fifth, at the end of each interview, major thematic areas were presented to the study participants for checking interpretations and comments. Sixth, an audit trail was conducted to verify the study findings.

## Results

### Socio-demographic characteristics

**Figure 1:**
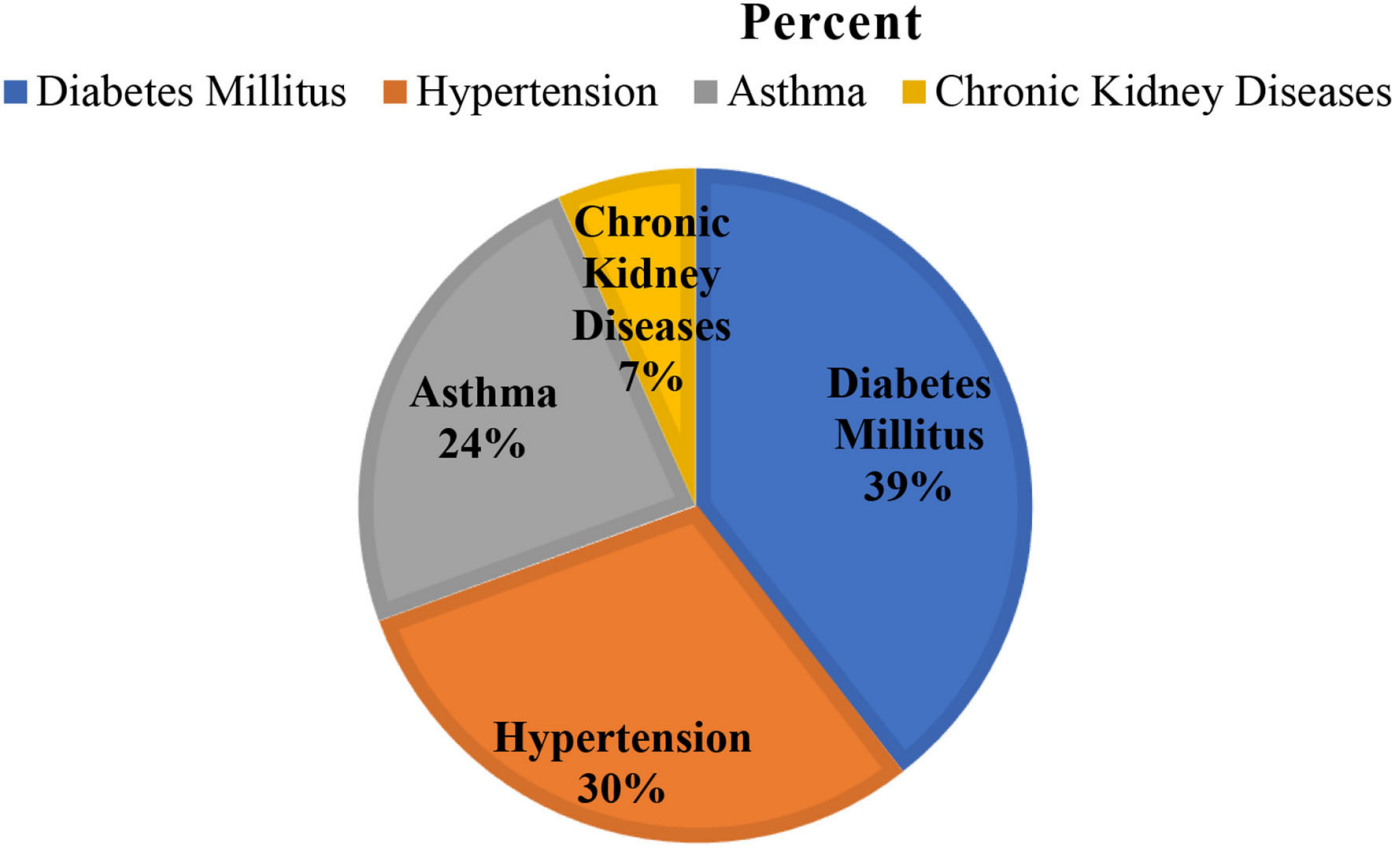
Chronic diseases of the participants at Jimma town 2021, Jimma, Ethiopia.

A total of 422 participants were interviewed giving a response rate of 100%. The majority of 266 (63%) respondents were men. The mean age of the respondents was 37.20 ± 13.14 years with a minimum age of 18 and a maximum of 84 years. Merchant and governmental employees accounted for 158 (37.4) and 150 (35.5%), respectively (Table 2). Nearly one-fifth (18.2%) of the participants had a chronic illness; among these, diabetes was found to be the most common disease followed by hypertension (Figure 1).

**Table 2:** Socio-demographic characteristics of participants at Jimma town 2021, Jimma, Ethiopia.

**Variables**	**Category**	**Number**	**Percentage**
Sex	Male	266	63.0
	Female	156	37.0
Age (years)	18–29	144	34.1
	30–39	125	29.6
	40–49	86	20.4
	≥50	67	15.9
Marital status	Married	282	66.8
	Single	112	26.5
	Others^*^	28	6.6
Religion	Muslim	145	34.4
	Orthodox	150	35.5
	Protestants	115	27.3
	Others^†^	12	2.8
Educational status	Unable to read & write	66	15.6
	Primary	97	23.0
	Secondary	90	21.4
	College and above	169	40.0
Occupational status	Merchant	158	37.4
	Governmental employee	150	35.5
	Non-governmental employee	71	16.8
	Others^‡^	43	10.2
Family size	<3	206	48.8
	3–5	137	32.5
	≥5	79	18.7
Average monthly income (ETB)	<4000	232	55.0
	4000–5999	64	15.2
	≥6000	126	29.9
Live with COVID19 risk population	Yes	225	53.3
	No	197	46.7
Having chronic illness	Yes	77	18.2
	No	345	81.8

* Widowed, divorced, separated;

† catholic, Wakeffata;

‡ retired, daily labor, housewife.

Of the total 77 respondents with chronic diseases, diabetes mellitus (39%) and hypertension (30%) were the most common (Figure 1).

Different interesting findings were found in the study. The qualitative findings indicated that the community had a problem with COVID-19 risk perceptions. However, problems related to risk perceptions had disparity among the populations and it been described below.

### COVID-19 personal experience and vaccine uptake

**Figure 2:**
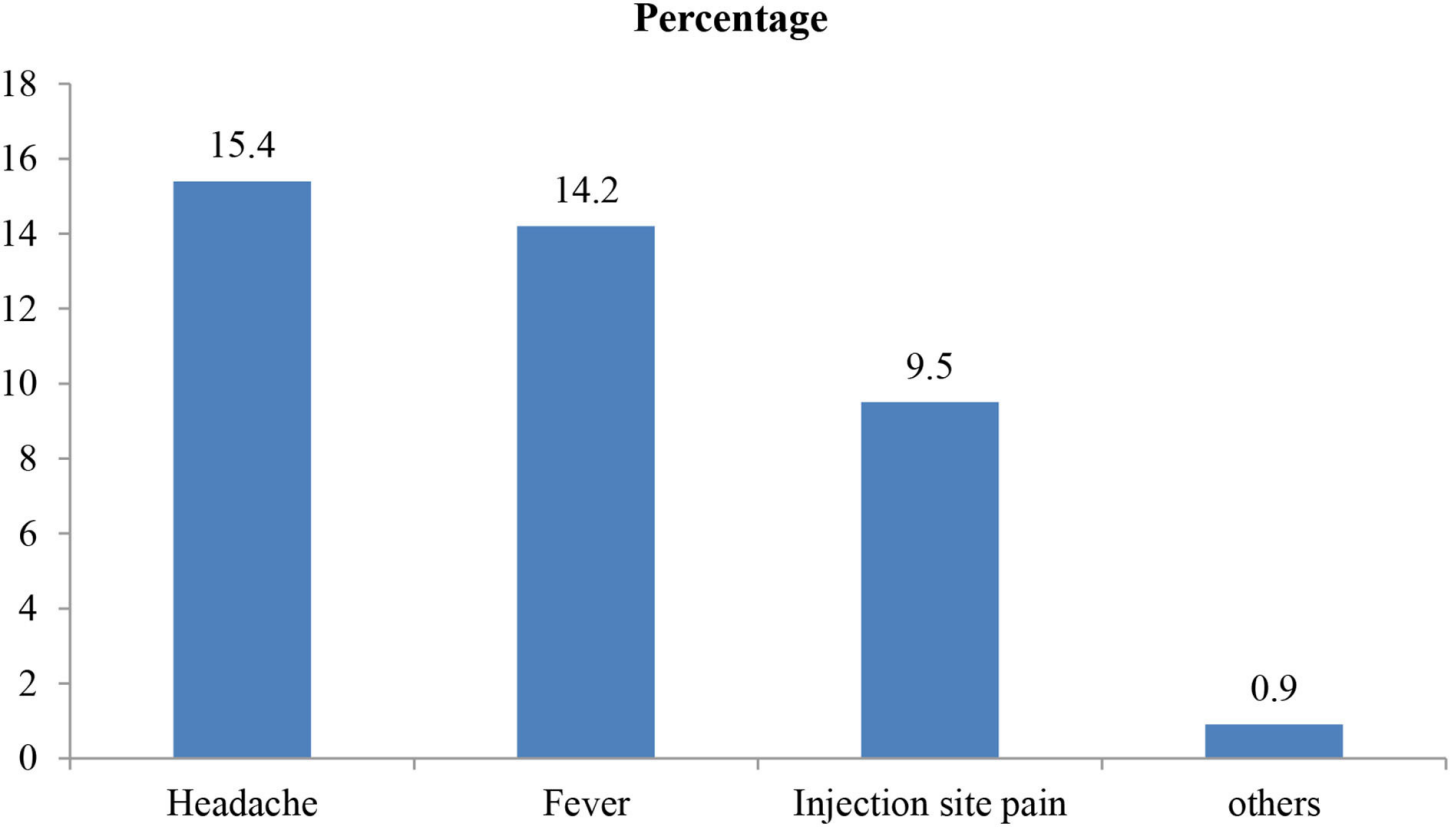
Experienced side effects of vaccine the participants at Jimma town 2021, Jimma, Ethiopia.

Of a total of study participants, 74 (17.5%) of them had a history of COVID-19 infection. Similarly, about 152 (36.0%) of them had known to socially (nearby people) infect people with the infection. Regarding experience on the outcome of infected people, 64 (15.2%) and 34 (8.1%) who responded to the infection improved and died, respectively. About 147 (34.8%) of the study participant had taken the COVID-19 vaccine, of which 84 (57%) participants had experienced side effects such as headache, fever, and injection site pain (Figure 2).

The reasons for not taking the COVID-19 vaccine were assessed using multiple answers. Those who did not take the COVID-19 vaccine mentioned reasons such as no chance of contracting the infection [142 (33.6%)], fear of side effects [120 (28.4%)], preferring other ways of protection [114 (27.0%)], prior history of adverse reaction to any vaccine [49 (11.6%)], and perception that the vaccine itself causes infection [29 (6.9%)] (Table 3).

**Table 3:** COVID-19 personal experience and Vaccine uptake of participants at Jimma town 2021, Jimma, Ethiopia.

**Variable**	**Category**	**n**	**%**
Infected with COVID-19	Yes	74	17.5
	No	348	82.5
Social infected with COVID-19	Yes	152	36.0
	No	270	64.0
Outcome	Improved	64	15.2
	Died	34	8.1
	don't know	14	3.3
	Total	112	26.5
Took vaccine	Yes	147	34.8
	No	275	65.2
Experienced side effects	Yes	84	19.9
	No	63	14.9
Reasons for not taking	No chance	142	33.6
	Fear of SE	120	28.4
	Prefer other ways of protection	114	27.0
	Prior adverse reaction to any vaccine	49	11.6
	Vaccine causing COVID-19	29	6.9

Similarly, the qualitative findings support these findings. It was mentioned that there were community members who perceived that COVID-19 is a curse from God or Allah. There were also study participants who mentioned that they were not at risk of contracting the infection; perceiving that GOD protects our community.

“*I am not using a mask or other preventive measures. This is because am not at risk of contracting the infection. Also, I do not want to take the vaccine. This is because, first, am not at risk; second, I heard that it caused blood clotting, skin rash, redness of the eye, and fever. This is what I have observed and heard from those who took the vaccine.” (43 years old, female, community member).”*

The study participants also mentioned that there were community members who perceived that there was no COVID-19 infection or disease. For example, a 37-year- old man from the community mentioned that:

“*There is no COVID-19 infection. I do not know even someone infected or who died from COVID-19 infection in my society. Generally, it is a simple talk that is communicated from different sources, including higher officials.”*

Similarly, health workers who participated in this study also mentioned that the community did not wear a mask while seeking health care from the health facilities. For example, one health extension worker mentioned that the community perceived that there is no COVID-19 infection. In addition, they faced challenges while conducting home visitation and different meetings to educate them about the infection and its prevention mechanism, and also sensitize/promote the vaccine:

“*The community perceives that there is no COVID-19 infection. They are not volunteering to accept or take the vaccine. They tell as it is a disease that affects white peoples.”*

### COVID-19 prevention practices status

The descriptive analysis indicated that about 130 (30.8%) and 41 (9.7%) of the participants shake hands with others often and always, respectively. Approximately 222 (52.6%), 217 (51.4%), and 171 (40.5) of them often sanitized their hand with an alcohol-based sanitizer, washed hands for at least 20 s, and cleaned their hands before touching their face, respectively. However, 182 (43.1%) of them rarely maintained their social distance. Most importantly, approximately 100 (23.7%) and 169 (40.0%) of the participants wore masks rarely and often, respectively (Table 4).

**Table 4:** COVID-19 Preventive Practices of participants at Jimma town 2021, Jimma, Ethiopia.

**Variable**	**Responses N (%)**				
	**Never**	**Rarely**	**Sometimes**	**Often**	**Always**
Frequency of shake hands	111(26.3)	95(22.5)	45(10.7)	130(30.8)	41(9.7)
Frequency of sanitizing hand with alcohol-based sanitizer	29(6.9)	59(14.0)	65(15.4)	222(52.6)	47(11.1)
Frequency of washing hands with soap for 20 seconds	25(5.9)	78(18.5)	68(16.1)	217(51.4)	34(8.0)
Frequency of cleaning hands before touching face	19(4.5)	91(21.6)	115(27.3)	171(40.5)	26(6.2)
Frequency of covering face with elbow while coughing	26(6.2)	50(11.8)	92(21.8)	216(51.2)	38(9.0)
Frequency of avoiding touching eyes, nose and mouth before washing hands	32(7.6)	95(22.5)	110(26.1)	160(37.9)	25(5.9)
Frequency of avoiding going outdoor unnecessarily	82(19.4)	166(39.3)	84(19.9)	78(18.5)	12(2.8)
How often do you maintain a minimum distance of 1 meter from others?	76(18.0)	182(43.1)	73(17.3)	80(19.0)	11(2.6)
How often do you avoid social gatherings?	64(15.2)	170(40.3)	72(17.1)	105(24.9)	11(2.6)
How often do you wear a mask while going out of home?	28(6.6)	101(23.9)	68(16.1)	189(44.8)	36(8.5)
Frequency of ensuring as both mouth and nose covered while wearing mask	22(5.2)	100(23.7)	82(19.4)	169(40.0)	49(11.6)
Frequency of disposing mask in the dust bin after use	23(5.5)	116(27.5)	82(19.4)	169(40.0)	32(7.6)
How often do you clean your items with sanitizer when you come home?	23(5.5)	134(31.8)	91(21.6)	151(35.8)	23(5.5)
How often do you take precautions when buying things?	26(6.2)	133(31.5)	102(24.2)	140(33.2)	21(5.0)
How often do you obey the government restrictions regarding the COVID-19 pandemic?	26(6.2)	76(18.0)	142(33.6)	160(37.9)	18(4.3)

Similarly, the qualitative findings indicated that there was a disparity in applying the preventive measures among the community members, including the school community. It was mentioned that the community use face masks irregularly. They wore it while they went in the market area. However, utilize sometimes on transportation, or while walking outdoor for certain issues, events like weeding or burial ceremony, etc. for example, a 65-year-old female community member said:

“*The community perceives that there is no coronavirus infection. I irregularly use a face mask, because; it causes shortness of breath. Also, I perceive that there is no COVID infection. We protect ourselves through traditional medication like ginger and other home remedies. In addition, I don't want to take a vaccine. This is because I know a person died after taking the vaccine.”*

Similarly, it was mentioned that it is difficult to stay at home with our country's economy. This is because the community has children to serve, and everyone has to conduct activities every day to survive. They said that it is better to die eating rather than staying at home. For example, a 54-year-old female community member mentioned that:

“*We have children who go to school. They need a uniform, meal and other materials. Therefore, how can we serve them if we stay at home? I sell food items on this street to serve them. We wait for our death working our day-to-day activity rather than staying at home (kkkkk-laughing).”*

### Factors associated with COVID-19 preventive practices

#### Binary and multivariable analysis

Variables like age category, family size, occupation, living with COVID-19 high-risk population, suffering from chronic diseases, being infected with COVID-19, social infection with OVID-19, and taking of COVID-19 vaccine were associated with good prevention practices at a *p*-value of less than 0.25. The result of multivariable logistic regression indicated that age category, living with COVID-19 high-risk population, having chronic diseases, and taking the COVID-19 vaccine were independently associated with good preventive practices against COVID-19.

In this study, 56 (13.3%) of the study participants had adopted good prevention practices against COVID-19. Compared with younger people (<30 years), people ≥50 years of age were 3 times more likely to have adopt good preventive measures against COVID-19 [AOR = 2.85, 95%, CI = 1.246.53]. People recovering from COVID-19 were also found to be associated with good prevention practices against COVID-19. Participants who have recovered from COVID19 were 2 times more likely to have good COVID-19 prevention practices than their counterparts [AOR = 2.41, 95%, CI = 1.184.92]. The odds of having good COVID-19 prevention practices were 4 times higher among participants who had chronic diseases [AOR = 3.70, 95%, CI = 1.887–0.25] compared to their counterparties. The respondent living with high-risk COVID-19 populations were 3 times more odds likely to take good precautions against COVID-19 than their counterparties [AOR = 2.96, 95%, CI = 1,475–0.99] (Table 5).

**Table 5:** Bivariable and multivariable analysis of factors associated with COVID-19 prevention practice among respondents at Jimma town 2021, Jimma, Ethiopia.

**Variable**	**Category**	**Preventive practice**		**COR (95% CI)**	**AOR (95% CI)**
		**Poor (%)**	**Good (%)**		
Age (years)	18–29	130(30.8)	14(3.3)	1	1
	30–39	116(27.5)	9(2.1)	0.72[0.30–1.73]	0.58[0.23–1.45]
	40–49	75(17.8)	11(2.6)	1.36[0.59–3.15]	0.82[0.33–2.05]
	≥50	45(10.7)	22(5.2)	4.54[2.14–9.62]	2.85[1.24–6.53]^*^
Family size	<3	184(43.6)	22(5.2)	**1**	**1**
	3–5	117(27.7)	20(4.7)	1.43[0.75–2.73]	0.83[0.39–1.75]
	≥5	65(15.4)	14(3.3)	1.80[0.87–3.73]	0.82[0.33–2.01]
Occupational status	Merchant	142(33.6)	16(3.8)	1	1
	Governmental employee	129(30.6)	21(4.9)	1.45[0.72–2.89]	1.9[0.87–4.17]
	NGO employee	59(13.9)	12(2.8)	1.81[0.81–4.05]	1.75[0.71–4.36]
	Others^‡^	36(8.5)	7(1.7)	1.73[0.66–4.51]	1.32[.45–3.89]
Live with under 18/65+	Yes	182(43.1)	43(10.2)	3.34[1.74–6.43]^**^	2.96[1.47–5.99]^*^
	No	184(43.6)	13(3.1)	1	1
Chronic illness	Yes	50(11.8)	27(6.4)	5.88[3.22–10.76]^**^	3.70[1.88–7.25]^**^
	No	316(74.9)	29(6.9)	1	1
Infected with COVID−19	No	311(73.7)	37(8.8)	1	1
	Yes	55(13.0)	19(4.5)	0.34[0.19–0.64]^*^	2.41[1.18–4.92]^*^
Friends infected with COVID−19	No	244(57.8)	26(6.2)	1	1
	Yes	122(28.9)	30(7.1)	2.31[1.31–4.07]^*^	1.22[0.62–2.39]
Took vaccine	Yes	121(28.7)	26(6.2)	1	1
	No	245(58.1)	30(7.1)	0.57[0.32–1.01]	0.64[0.34–1.22]

* <0.05;

** <0.01;

‡ retired, daily labor, house wife.

## Discussion

Out of 422 participants, only 56 (13.3%) of the participants had good COVID-19 prevention practices. This implies that the majority of community members did not adhere to the COVID-19 preventive practices recommended by the Ministry of Health or WHO. This finding is comparable with a study conducted in Qellam Wallaga (10%) (15). However, it is lower than the findings of an online survey conducted among educated people and health professionals in different countries, including Ethiopia (16–22). These variations might be brought about by variations in socioeconomic level, the healthcare system, research design, selection standards, educational status, and professions. These highlights the necessity of developing health education programs and carrying out communication interventions for social and behavioral changes. This is because the qualitative finding indicated that there were misconceptions related to the infection and prevention practices that need such interventions.

People aged 50 years and above were one of the predictors of good prevention practices for COVID-19. This implies that there was a disparity among different age groups of the community members in practicing the preventive measures. However, the infection affects all segments of the population, even if aged; particularly, people with chronic illnesses are more at risk for the infection. Therefore, this also underscores that there is a need to conduct social and behavioral change communication interventions that target all community members regardless of their age category to adhere to the preventive measures. Similarly, this finding was supported by a study conducted among educated persons and health professionals in Peru and Ethiopia (17–19). On the other hand, the present finding was contrary to the study performed in Egypt which revealed that young age was a good protective age (20). This variation could be that the older adults feared COVID-19 as they are at a high risk of contracting the disease. So, they strictly followed the recommendation of WHO's COVID-19 prevention practices. Furthermore, this difference might be due to the socio-economic condition, health care system, study design, selection criteria, and educational status.

Recovery from COVID-19 infection has a statistically significant association with good COVID-19 prevention practices. This might imply that people already infected with the infection knew more about its impact (i.e., social, psychological, economic, health, etc.) and therefore, protected themselves as they have a certain experience. This indicated that there was a disparity among the community members related to exposure to the infection. However, this does not mean that they are the only people affected by the infection; it is obvious that all people are at risk to develop the infection. Therefore, this underscores a need to design health education programs and conduct similar interventions as was said earlier to avoid this disparity and make all people adhere to the preventive measures, thereby controlling the infection. This study was in harmony with the online survey conducted among educated Ethiopians (18) and a cohort study done in Ethiopia (21). The association might be explained by studying the people who recovered from COVID-19 infection who had adapted to a variety of coping mechanisms like enhancing standard hygiene, continuing to wear masks, practical social distancing, and using different media platforms to communicate with friends, and family to decrease the spread of COVID-19 infection. The present study disagrees with a study done in Western Ethiopia (22). This might be due to too much conflicting information on vaccines, misinformation, rumors, and disinformation on COVID-19 that have a potential impact on people's knowledge and attitude, which may further create a conflict on the preventive measures to be taken (23).

This study also showed that people living with a high risk of COVID-19 (over 65 years) had taken good preventive measures. This implies that community members might adhere to the preventive practices while staying out of this segment of the population age range. However, even if there might be a difference in the level of risk, all peoples are at risk of COVID-19 infection. Therefore, this underscores a need to design a health education program and conduct a risk communication intervention to make all people adhere to the preventive measures regardless of the age of their household or outdoor contact. This study was in line with a study conducted, in Addis Ababa, Ethiopia, among health care professionals (24). But, this study was incomparable to a study conducted in the Amhara region, Ethiopia (25). This discrepancy might be due to the differences in sociodemographic characteristics and study participants involved.

Suffering from a chronic disease was also among the factors that are associated with good COVID-19 prevention practices. This finding was supported by a study conducted in Dire Dawa (26), in the Amhara region of Ethiopia and the Gedeo zone of southern Ethiopia (25, 27). A possible explanation is that people with chronic diseases have a better chance of taking care of themselves regularly because they are close to a health professional (28). However, it does not mean that only these peoples are at risk of COVID-19 infection or could die from the disease. From our experience, many young people without chronic illnesses were exposed to the infection or died of the disease. Furthermore, the qualitative findings indicated that there were misconceptions about the people at risk of the disease, i.e., white people or rich people. Therefore, this calls for a need to conduct similar interventions suggested above to change the community's perception, adhere to the preventive measures, and prevent the spread of the infection.

### Limitation of the study

The results of this study are based on self-reported data, and respondents may report socially acceptable overestimations or underestimations of the responses rather than true or sincere responses. Additionally, the design effect was not considered during sample size calculation even though a 10% non-response rate was added. Despite the above limitations, the present finding has a strong point, that is this study was community-based and triangulated with the qualitative findings. Moreover, the results obtained in this finding provide important information to reinstate COVID-19 prevention practices in the community.

## Conclusion and recommendation

In this study, it was understood that participants had poor COVID-19 preventive practices. There was a disparity in adhering to the preventive practices in relation to (i.e., 50 and above years) the experience of contracting COVID-19, a high-risk group (i.e., under 18 and above 65 years old), and having a chronic disease. In addition, the community had different misconceptions or risk perceptions related to COVID-19 infection and preventive practices. This highlights the need to design health education programs and implement risk and/or social and behavior change communication interventions to change perceptions or misconceptions of people or community members to bring about the desired behavior change and prevent the spread of COVID-19. Furthermore, researchers, local health planners, and other stakeholders should conduct a large-scale study.

## Data availability statement

The original contributions presented in the study are included in the article/supplementary material, further inquiries can be directed to the corresponding author/s.

## Ethics statement

The studies involving human participants were reviewed and approved by Institutional Review Board of Jimma University. The patients/participants provided their written informed consent to participate in this study.

## Author contributions

DB, KT, and DA: conceptualization, data curation, formal analysis, project administration, validation, visualization, and writing the original draft. KT, DB, BL, DA, AD, UG, KK, NB, AL, GK, and AN: methodology, writing, reviewing, and editing. All authors read and approved the final version of the manuscript.

## Funding

This research was funded by the Research and Postgraduate Coordinating Office of Jimma University.

## Conflict of interest

The authors declare that the research was conducted in the absence of any commercial or financial relationships that could be construed as a potential conflict of interest.

## Publisher's note

All claims expressed in this article are solely those of the authors and do not necessarily represent those of their affiliated organizations, or those of the publisher, the editors and the reviewers. Any product that may be evaluated in this article, or claim that may be made by its manufacturer, is not guaranteed or endorsed by the publisher.
